# The Impact of Opium Consumption on Blood Glucose, Serum Lipids and Blood Pressure, and Related Mechanisms

**DOI:** 10.3389/fphys.2016.00436

**Published:** 2016-10-13

**Authors:** Hamid Najafipour, Ahmad Beik

**Affiliations:** ^1^Cardiovascular Research Center and Department of Physiology, Institute of Basic and Clinical Physiology Sciences, Kerman University of Medical SciencesKerman, Iran; ^2^Physiology Research Center, Kerman University of Medical SciencesKerman, Iran

**Keywords:** opium, blood glucose, serum lipids, blood pressure, diabetes, dyslipidemia, hypertension, prevalence

## Abstract

**Aim:** Substance abuse has become a universal crisis in our modern age. Among illegal substances, opium and its derivatives have been ranked second in terms of usage after cannabis in the world. In many Asian regions, the use of opium enjoys a high social acceptance; hence, some common people and even medical practitioners believe that opium lowers blood glucose and pressure and treat dyslipidemia. How much this belief is scientifically justified?

**Method:** The results of available studies on both humans and animals searched in different search engines up to mid-2016 were integrated (78 articles). Upon the findings we try to offer a more transparent picture of the effects of opium on the mentioned factors along with the probable underlying mechanisms of its action.

**Results:** Taken together, a variety of evidences suggest that the consumption of opium has no scientific justification for amendment of these biochemical variables. The mechanisms proposed so far for the action of opium in the three above disorders are summarized at the end of the article. Short term effects seems to be mostly mediated through central nervous system (neural and hormonal mechanisms), but long term effects are often due to the structural and functional alterations in some body organs.

**Conclusion:** Although opium may temporarily reduce blood pressure, but it increases blood glucose and most of blood lipids. Moreover its long term use has negative impacts and thus it aggravates diabetes, dyslipidemia and hypertension. Accordingly, it is necessary to inform societies about the potential disadvantages of unauthorized opium consumption.

## Introduction

Substance abuse is today's serious health and socio-economic issue (United Nations Office on Drugs and Crime, 2014). Globally, after cannabis, opioids have the highest rate of illicit drug consumption. United Nations' Office on Drugs and Crime estimated that in 2013, 32.4 million of the world's adult population consumed opioids. Of these, 16.5 million were users of opiates (opium and its derivatives specifically heroin). Asia is the most important market for opiates, and Asian countries have monopolized about two thirds of the opiates market (United Nations Office on Drugs and Crime, 2015). One of the reasons for high usage in these countries might be the easy access to the drug because the main opium producer countries such as Afghanistan, and to a less extent, Myanmar, and Laos are located in this continent. Some Asians believe that opium could have positive impacts on lowering of serum lipids, blood glucose and blood pressure. Such a belief has some adherents even among medical practitioners (Farahani et al., 2008; Jafari et al., 2009). Is this ancient belief scientifically justified? Over the recent years many studies have been performed on humans and animals to answer this question or have reviewed its effects on cardio-metabolic diseases (Masoudkabir et al., 2013). The objective of present review is to collect and integrate the newest information with previous findings to clarify the effects of opium on forenamed biochemical variables and the underlying mechanisms of the action of opium on these conditions. In this regards and in addition to the studies on blood glucose, blood pressure and serum lipids, the effects of opium on the prevalence of hypertension, dyslipidemia and diabetes, have also been included. Furthermore, since morphine is the main alkaloid constituent of opium (see below), and many physiological and pharmacological effects of opium are ascribed to morphine, few studies that focus on the impact of morphine on these disorders are also considered.

Overall the results of animal studies may be more consistent and reliable because there are less confounding variables in these studies to affect the results. Therefore, we included the results of the related animal studies in the review. Some of these variables that may alter the effect of opium in human include, sex and age range of subjects, nutritional factors, psycho-social conditions, physical activity level, concomitant use of other substances such as alcohol, cigarette, tobacco, and underlying diseases. Therefore, in this review we firstly address the results of human studies and then proceed to the results on the animal studies and related mechanisms.

## The origin and ingredients of opium and their pharmacology

Primary opium is a milky latex extracted by scoring unripe capsules of opium poppy plant (*Papaver somniferum)*. After being exposed to air, it turns into a dark brown and solidified substance (Schiff, 2002). In addition to water, various sugars and several organic acids, around 50 alkaloids have also been isolated from opium so far and the latter constitute approximately 20–30% of its raw weight (Schiff, 2002; Najafipour et al., 2010). Alkaloids are categorized to two main groups (Phenanthrenes and Benzylisoquinolines) and several minor groups (Schiff, 2002; European Food Safety Authority, 2011). Phenanthrenes including morphine, codeine, and thebaine constitute the predominant psychoactive ingredients (European Food Safety Authority, 2011). Phenanthrenes and opium derived semisynthetic drugs such as heroin are termed opiates. All morphine like analgesics, regardless of their source which encompass opiates, endogenous opioid peptides and synthetic opioids constitute the drug class named opioids (Vuong et al., 2010). Papaverine relaxes smooth muscle and noscapine has an antitussive property (Kalant, 1997; European Food Safety Authority, 2011). Morphine is both the most active and the most copious alkaloid in opium with codeine being in the second order (Schiff, 2002; European Food Safety Authority, 2011). Opioids act via at least three types of receptors, named mu (μ), kappa (κ), and delta (δ) (Vuong et al., 2010). Mostly, opioid μ-receptors mediate the effects of morphine. Euphoric and analgesic properties are the most common reasons for the consumption of opium by its users (Kalant, 1997).

In medical use opioids are essential in the management of myocardial infarction, severe pain subsequent to surgical procedures, late-stage cancers, and other torturous diseases and conditions. Hence, availability of these substances for medical purposes should be ensured and their misuse should be prevented (United Nations Office on Drugs and Crime, 2014).

The consumption of opium for medical and recreational purposes has a long history. For inhalation, opium is directly heated by charcoal or a hot and thin metal rod until the active alkaloids, particularly morphine, are smoked. During smoking, a great deal of morphine in the opium is lost. In the oral method, the bioavailability of morphine is diminished due to the metabolism in the digestive system and the hepatic first pass effect, but codeine is less affected (Kalant, 1997; European Food Safety Authority, 2011). Furthermore, morphine is slowly absorbed into the bloodstream in the small intestine, thus the action of oral opium consumption starts after a delay, and the duration of this action is long. On the contrary, after smoking opium, the onset of action is fast and intense, and the duration of the action is shorter (Kalant, 1997).

## The impact of opium on blood glucose and diabetes

### Human studies

Most clinical reports imply that opium has no remarkable impact on blood glucose (Table 1) whether in non-diabetic (Asgary et al., 2008; Roohafza et al., 2013; Javadi et al., 2014; Masoomi et al., 2015; Sanli et al., 2015) or in diabetic individuals (Karam et al., 2004; Hosseini et al., 2011; Rezvanfar et al., 2011; Najafi and Sheikhvatan, 2012a; Bayani et al., 2014; Rahimi et al., 2014). In some of these studies, glycated hemoglobin (HbA1c), as an indicator of poor control of blood glucose, was significantly higher in addicted groups (Karam et al., 2004; Asgary et al., 2008). Another report on the addicted subjects attempting opium abstention showed that fasting blood glucose (FBS) did not change considerably 3 months after opium withdrawal (Mahmoodi et al., 2012). A study on heroin and methadone addicts indicated that FBS was similar with control group, but insulin was significantly higher in the addicted groups. After oral or intravenous glucose load, plasma glucose was significantly higher and insulin response was significantly lower in the addicted groups. Authors suggested that insulin response may be impaired either due to the functional failure of stressed β-cells or by altering of its action on target tissues (Ceriello et al., 1987). In another research, after the subjects had a standard meal, FBS was not different between the heroin addicts and the control group. C-peptide immunoreactivity, however, was lower and insulin immunoreactivity was higher in the addicts. It was concluded that heroin addiction may cause β-cell failure and contemporaneously produce hyperinsulinemia due to the alteration of the rate of hepatic extraction of insulin (Zandomeneghi et al., 1988). In one study in opium addicts, FBS was significantly higher compared to that of the non-addicts (Gozashti et al., 2015). In this report, the increase of FBS was accompanied with a significant decrease in the fasting blood insulin.

Table 1**The studies indicating effects of opium on blood glucose and diabetes**.

**Table:** 

**A—HUMAN STUDIES**								
**Study reference**	**Type of substance and duration of usage**	**Type of study**	**Study population**	**Underlying disease**	**Results (addicts compared to non-addicts)**			
					**BMI**	**FBS**	**HbA1c**	**Prevalence of diabetes**
Asgary et al., 2008	Opium addicted	Case-control	*n* = 720 smoker men (360 opium addict, 360 non-addict)	None	ND	↔	↑	ND
Sanli et al., 2015	Opium addicted	Case-control	*n* = 115 men/women (46 opium dependent, 69 non-dependent)	None	ND	↔	ND	ND
Masoomi et al., 2015	Opium addicted	Cross-sectional	*n* = 217 men (103 opium addict, 114 non-addict)	None	ND	↔	ND	↔
Roohafza et al., 2013	Opium addicted	Cohort	*n* = 569 men (99 opium addicts, 470 non-addicts)	AMI	↔	↔	ND	ND
Javadi et al., 2014	Opium addicted	Cross-sectional	*n* = 304 men/women (152 opium user, 152 non-user)	AMI	ND	↔	ND	↔
Bayani et al., 2014	Opium (for more Than 6 months)	Cross-sectional	*n* = 97 men/women (48 opium addicts, 49 non-addicts)	ACS + type 2 diabetes	↔	↔	↔	Both groups were diabetic
Najafi and Sheikhvatan, 2012a	Opium addicted	Cross-sectional	*n* = 232 (26 opium addicts, 206 non-addicts)	advanced CAD+ type 2 diabetes	↓	↔	↔	Both groups were diabetic
Karam et al., 2004	Opium (for at least 1 year)	Case-control	*n* = 98 men/women (49 opium addict, 49 non-addict)	Type 2 diabetes	↔	↔	↑^#^	Both groups were diabetic
Hosseini et al., 2011	Opium (for more than 3 month)	Cross-sectional	*n* =456 men/women (228 opium addict, 228 non-addict)	Diabetes mellitus	↓	↔	ND	Both groups were diabetic
Rahimi et al., 2014	Opium addicted	Cross-sectional	*n* = 374 men/women (179 opium addict, 195 non-addict)	Type 2 diabetes	↓	↔	↔	Both groups were diabetic
Rezvanfar et al., 2011	Opium addicted	Cross-sectional	*n* = 232 men (88 opium user, 144 non-user)	Type 2 diabetes	↔	↔	↓	Both groups were diabetic
Ceriello et al., 1987	Heroin (for 2–7 years) Methadone (for 1–3 years)	Case-control	*n* = 45 men (15 heroin addict, 15 methadone treatment, 15 normal)	None	↔	↔	ND	ND
Zandomeneghi et al., 1988	Heroin (for at least 2 years)	Case-control	*n* = 20 men/women (10 heroin addict, 10 normal)	None	↔	↔	ND	ND
Gozashti et al., 2015	Opium addicted	Case-control	*n* = 108 men/women (53 opium addict, 55 non-addict)	None	↔	↑	ND	ND
Azod et al., 2008	Opium addicted	Case-control	*n* =69 men (23 opium addict, 46 non-addict)	Type 2 diabetes	↔	↓	↔	Both groups were diabetic
Afarinesh et al., 2014	Opium (for more than 2 years)	Cross-sectional	*n* = 320 men (90 opium dependent, 120 opium withdrawal, 110 healthy)	None	ND	↓	ND	ND
Shirani et al., 2010	Opium addicted	Cross-sectional	*n* = 939 men (opium addicts, and non-addicts)	CAD	↓	↓	↑	↓
Masoomi et al., 2008	Opium addicted	Cross-sectional	*n* = 240 men/women (126 opium addict, 114 non-addict)	AMI	ND	↓	ND	↓
Aghadavoudi et al., 2015	Opium (for 12.6 ± 7.7 years)	Cross-sectional	*n* = 325 men/women (117 opium addict, 208 non-addict)	CAD	↓	↓	ND	↓
Dehghani et al., 2013	Opium addicted	Cross-sectional	*n* = 460 men/women (239 opium addict, 221 non-addict)	AMI	ND	↓	ND	↓
Divsalar et al., 2010	Opium, Heroin	Cross-sectional	*n* = 112 men (42 opium addict, 35 heroin addict, 35 non-addict)	None	↔	↓^*^	ND	ND
Najafi and Sheikhvatan, 2012b	Opium addicted	Cross-sectional	*n* = 268 men/women (38 opium addict, 230 non-addict)	CAD	↓	ND	ND	↓
Yousefzadeh et al., 2015	Opium addicted	Cross-sectional	*n* = 5332 men/women (811 current opium users, 176 former opium users. 4340 non-user)	None	↓	ND	ND	↑
Davoodi et al., 2005	Opium addicted	Cohort	*n* = 160 (45 opium-dependent, 115 non-dependent)	AMI	ND	ND	ND	↔
Najafipour et al., 2015a	Opium addicted	Cross-sectional	*n* = 5896 men/women (non-users, occasional users, dependent users)	normal/T2DM	↔	ND	↔	↔

**Table:** 

**B—ANIMAL STUDIES**						
**Study reference**	**Animal**	**Type of substance and duration of usage**	**Study population**	**Underlying disease**	**Results (addicts compared to non-addicts)**	
					**FBS**	**HbA1c**
Mohammadi et al., 2012	Mouse	Opium (orally for 1 month)	*n* = 16 (8 opium addict, 8 non-addict)	None	↔	ND
Sadeghian et al., 2009	Rat	Opium (orally for 1 month)	*n* = 20 male (10 addict, 10 non-addict)	Diabetes mellitus	↔	↔
Mami et al., 2011	Rabbit	Opium (orally for 60 days)	*n* = 40 (20 addict, 20 non-addict)	None	↑	ND
Sadava et al., 1997	Rat	Methadone (orally for 35 days)	*n* = 70 female (35 addict, 35 control)	None	↑	ND

Symbols: ↓, decrease; ↑, increase; ↔, no difference; ^#^, addicted males compared to control males; ^*^, opium addicted compared to control.

FBS, fasting blood sugar; HbA1c, glycated hemoglobin; ND, not detected; None, no disease; AMI, acute myocardial infarction; CAD, coronary artery disease.

In contrast, several reports have indicated that opium has a positive impact on the fall of FBS. In a study on diabetic patients, the opium addiction significantly decreased FBS and 2HPP (glucose concentration 2-h post prandial) compared to the non-addicted patients, but HbA1c did not alter (Azod et al., 2008). The reduction of 2HPP might be due to the decrease of gastric emptying due to opioid μ-receptor activation and thereafter delaying the intestinal glucose absorption (Azod et al., 2008). Several studies on non-diabetic subjects also showed lower levels of FBS in opium addicts compared to the non-addicts (Masoomi et al., 2008; Shirani et al., 2010; Dehghani et al., 2013; Afarinesh et al., 2014; Aghadavoudi et al., 2015); yet, FBS in the heroin addicts was similar to that of the non-addicts (Divsalar et al., 2010).

The findings regarding the prevalence of diabetes are controversial as well (Table 1). Some studies have demonstrated the lower rate of diabetes among opium addicts compared to the non-addicts (Masoomi et al., 2008; Shirani et al., 2010; Najafi and Sheikhvatan, 2012b; Dehghani et al., 2013; Aghadavoudi et al., 2015). One study showed the opposite result (Yousefzadeh et al., 2015), while the data gathered from some other studies have shown no relation between opium addiction and the rate of diabetes mellitus (Davoodi et al., 2005; Javadi et al., 2014; Masoomi et al., 2015). In a more recent population-based study by the authors of this article on 5900 individuals, opium consumption had no significant association with diabetes (Najafipour et al., 2015a), yet the addicted people had a higher rate of uncontrolled diabetes (Najafipour et al., 2015b).

### Animal studies

In none of the animal studies, opium or opioids decreased blood glucose (Table 1). In one study on normoglycemic mice, there was no significant difference in the blood glucose between the opium addicted group and the control group (Mohammadi et al., 2012). Moreover, in one study on diabetic rats, regarding serum glucose, there was no significant difference between the opium treated and the control group (Sadeghian et al., 2009). Mami et al. showed that in opium addicted rabbits, serum FBS was significantly higher compared to that of the non-addicted ones (Mami et al., 2011). In one study on conscious dogs, intravenous infusion of morphine had dual effects, low dose (2 mg/h) did not change blood glucose, but high doses (8.16 mg/h) caused hyperglycemia subsequent to the elevation of glucose production in the liver and reduction of glucose clearance in peripheral tissues. These effects were attributed to the increased levels of epinephrine, glucagon, and cortisol in plasma (Radosevich et al., 1984). In a similar study done on conscious rabbits, the administration of 0.3 and 3 mg/kg morphine produced the same results. The authors suggested that hyperglycemia made by high doses might be a response to the secretion of adrenaline (May et al., 1988). In the study of Molina et al on rats, central infusion of morphine significantly raised the blood glucose. These data indicated that hyperglycemic effects of morphine were mediated through central nervous system (CNS), and were led to increased hepatic glucose production secondary to glycogenolysis and in part due to the decrease of insulin release (Molina et al., 1994). Likewise, single intravenous injections of morphine (0.5 mg/kg) resulted in more hyperglycemia in diabetic conscious dogs compared to the normal dogs. This effect of morphine was partly attributed to the increase of glucagon secretion (Ipp et al., 1980). Furthermore, in intra-peritoneally opium injected rats, blood glucose increased gradually between 30 and 120 min after the injection of opium, compared to that of the control group (Asadi et al., 2008). In one study, the orally methadone therapy in the rats for a 35-days period by increasing doses (0.5–1.8 mg/kg) raised blood glucose significantly. In this study, blood glucose was returned back to its normal level 30 days after the withdrawal. In addition, while being exposed to methadone, the animals showed impairment in glucose tolerance test (Sadava et al., 1997).

## The impact of opiumonlipid profile and dyslipidemia

### Human studies

There is a disparity regarding the impact of opium on lipid indices in humans (Table 2). Several studies on healthy subjects and on individuals who suffered from diabetes mellitus, acute myocardial infarction (AMI) and coronary artery disease (CAD) have indicated no significant relations between opium addiction and triglyceride (TG), total cholesterol (TC), low density lipoprotein (LDL), and high density lipoprotein (HDL) levels (Azod et al., 2008; Shirani et al., 2010; Najafi and Sheikhvatan, 2012a; Roohafza et al., 2013; Afarinesh et al., 2014; Bayani et al., 2014; Javadi et al., 2014; Masoomi et al., 2015; Sanli et al., 2015).

Table 2**The studies indicating the effects of opium on blood lipids and dyslipidemia**.

**Table:** 

**A—HUMAN STUDIES**										
**Study reference**	**Type of Substance and duration of usage**	**Type of study**	**Study population**	**Underlying disease**	**Results (addicts compared to non-addicts)**					
					**BMI**	**TG**	**TC**	**LDL**	**HDL**	**Prevalence of dyslipidemia**
Asgary et al., 2008	Opium addicted	Case-control	*n* =720 smoker men (360 opium addict, 360 non-addict)	None	ND	↔	↔	↔	↓	ND
Sanli et al., 2015	Opium addicted	Case-control	*n* = 115 men/women (46 opium dependent, 69 non-dependent)	None	ND	↔	↔	↔	↔	ND
Masoomi et al., 2015	Opium addicted	Cross-sectional	*n* = 217 men (103 opium addict, 114 non-addict)	None	ND	↔	↔	ND	ND	ND
Roohafza et al., 2013	Opium addicted	Cohort	*n* = 569 men (99 opium addicts, 470 non-addicts)	AMI	↔	↔	↔	↔	↔	ND
Javadi et al., 2014	Opium addicted	Cross-sectional	*n* = 304 men/women (152 opium user, 152 non-user)	AMI	ND	↔	↔	↔	↔	↔
Bayani et al., 2014	Opium (for more Than 6 months)	Cross-sectional	*n* = 97 men/women (48 opium addicts, 49 non-addicts)	ACS + type 2 diabetes	↔	↔	↔	↔	↔	↔
Najafi and Sheikhvatan, 2012a	Opium addicted	Cross-sectional	*n* = 232 (26 opium addicts, 206 non-addicts)	advanced CAD+ type 2 diabetes	↓	↔	↔	↔	↔	↔
Azod et al., 2008	Opium addicted	Case-control	*n* =69 men (23 opium addict, 46 non-addict)	Type 2 diabetes	↔	↔	↔	↔	↔	ND
Afarinesh et al., 2014	Opium (for more than 2 years)	Cross-sectional	*n* = 320 men (90 opium dependent, 120 opium withdrawal, 110 healthy)	None	ND	↔	↔	ND	ND	ND
Karam et al., 2004	Opium (for at least 1 year)	Case-control	*n* = 98 men/women (49 opium addict, 49 non-addict)	Type 2 diabetes	↔	↔	↔	ND	↓^#^	ND
Rahimi et al., 2014	Opium addicted	Cross-sectional	*n* = 374 men/women (179 opium addict, 195 non-addict)	Type 2 diabetes	↓	↔	↔	↔	↓	↓_h_
Salman et al., 2010	Opium addicted	Cross-sectional	*n* = 53 men/women (29 opium addict, 24 non-addict)	None	ND	↑	↑	↑	↔	ND
Aghadavoudi et al., 2015	Opium (for 12.6 ± 7.7 years)	Cross-sectional	*n* = 325 men/women (117 opium addict, 208 non-addict)	CAD	↓	↑	↔	↑	ND	↑
Maccaria et al., 1991	Heroin addicted	Case-control	*n* = 83 men/women (60 addict, 23 non-addict)	None	↔	↑	↓	ND	↓	↓^h^_c_
Gozashti et al., 2015	Opium addicted	Case-control	*n* = 108 men/women (53 opium addict, 55 non-addict)	None	↔	↔	↓	ND	↓	ND
Hosseini et al., 2011	Opium (for more than 3 month)	Cross-sectional	*n* =456 men/women (228 opium addict, 228 non-addict)	Diabetes mellitus	↓	↓	↔	↔	↔	↔
Rezvanfar et al., 2011	Opium addicted	Cross-sectional	*n* = 232 men (88 opium user, 144 non-user)	Type 2 diabetes	↔	↓	↔	↔	↔	ND
Davoodi et al., 2005	Opium addicted	Cohort	*n* = 160 (45 opium-dependent, 115 non-dependent)	AMI	ND	↓	↔	↔	↔	ND
Masoomi et al., 2008	Opium addicted	Cross-sectional	*n* = 240 men/women (126 opium addict, 114 non-addict)	AMI	ND	↓	↔	↔	↔	ND
Dehghani et al., 2013	Opium addicted	Cross-sectional	*n* = 460 men/women (239 opium addict, 221 non-addict)	AMI	ND	↓	↔	↔	↔	ND
Fatemi et al., 2008	Opium addicted	Case-control	*n* = 175 men (100 opium addict, 75 non-addict)	None	↓	↔	↓	↔	↔	ND
Divsalar et al., 2010	Opium, Heroin	Cross-sectional	*n* = 112 men (42 opium addict, 35 heroin addict, 35 non-addict)	None	↔	↔	↔	ND	ND	ND
Akif et al., 2013	Opium, Heroin addicted	Case-control	*n* =125 men (50 opium addict, 50 heroin addict, 25 non-addict)	None	ND	↔^*^	↑^*^	↑^*^	↔	ND
						↓_&_	↓_&_	↔_&_		
Najafipour et al., 2015a	Opium addicted	Cross-sectional	*n* = 5896 men/women (non-users, occasional users, dependent users)	None	↔	ND	ND	ND	ND	↔
Najafi and Sheikhvatan, 2012b	Opium addicted	Cross-sectional	*n* = 268 men/women (38 opium addict, 230 non-addict)	CAD	↓	ND	ND	ND	ND	↓
Yousefzadeh et al., 2015	Opium addicted	Cross-sectional	*n* = 5332 men/women (811 current opium users, 176 former opium users. 4340 non-user)	None	↓	ND	ND	ND	ND	↑

**Table:** 

**B—ANIMAL STUDIES**								
**Study reference**	**Animal**	**Type of Substance and duration of usage**	**Study population**	**Underlying disease**	**Results (addicts compared to non-addicts)**			
					**TG**	**TC**	**LDL**	**HDL**
Bryant et al., 1987	Rat	75 mg morphine pellet (5-days period)	Male rats (morphine group, sham group)	Hypercholesterolemia	↔	↑	↑	↓
								↔_n_
Mami et al., 2011	Rabbit	Opium (orally for 2 months)	*n* = 40 (20 addict, 20 non-addict)	None	↑	↑	↑	↔
Mohammadi et al., 2013	Hamster	Opium (orally for 1 month)	*n* = 24 male (6 control, 6 opium addict, 6 alcoholic, 6 opium addict and alcoholic)	None	↑_b_	↔	↑_b_	↓_b_
Najafipour et al., 2010	Rabbit	Opium (smoking for 3 days or 28 days)	*n* = 40 (7 control, 7 isoproterenol, 6 short-term opium, 6 short-term opium + isoproterenol, 6 long-term opium, 6 long-term opium + isoproterenol)	Hypercholesterolemia	↔	↔	↔	↓_r_
Sadeghian et al., 2009	Rat	Opium (orally for 1 month)	*n* = 20 male (10 addict, 10 non-addict)	Diabetes mellitus	↔	↔	ND	↔
Mohammadi et al., 2009	Rabbit (male)	Opium (orally for 3 months)	*n* = 28 (7 control, 7 hypercholesterolemic, 7 addicted, 7 hypercholesterolemic-addicted	None	↑^Y^	↑^Y^	↑^Y^	↔
					↔^n^	↓^n^	↓^n^	
Mohammadi et al., 2012	Mouse	Opium (orally for 1 month)	*n* = 16 (8 opium addict, 8 non-addict)	None	↔	↓	ND	↔

Symbols: ↓, decrease; ↑, increase; ↔, no difference; #, addicted males compared to control males; ^*^, opium addicted compared to control; &, heroin addicted compared to control; n, normocholesterolemia compared to control; y, hypercholesterolemia compared to control; r, chronic opium compared to control; B, both opium addict and alcoholic compared to control; H, HDL; c, total cholesterol.

ND, not detected; AMI, acute myocardial infarction; CAD, coronary artery disease; ACS, acute coronary syndrome; BMI, body mass index.

On the other hand, some studies have shown that opium addiction has a harmful effect on one or more lipid parameters. One study on diabetic patients indicated that HDL significantly decreased, or prevalence of low HDL increased in opium addicted compared to non-addicted patients. Yet, other lipids were not different between the two groups (Rahimi et al., 2014). In another study on diabetic patients, HDL was diminished only in men not in women (Karam et al., 2004). Asgary et al. who carried out a study on male cigarette smokers also had the same results. They showed that the ways of opium consumption (smoking or ingestion) had no impact on its outcomes; however, the duration of addiction of more than 2 years significantly augmented LDL (Asgary et al., 2008). Furthermore, in a study on patients who were candidates for coronary artery bypass grafting, the levels of LDL and TG were significantly higher in opium addicts than non-addicts (Aghadavoudi et al., 2015). In another study, it has been shown that the serum levels of TG, TC, and LDL in opium addicted individuals were significantly higher (Salman et al., 2010) or heroin addicts had lower TC and TG, and higher VLDL than the non-addicts (Akif et al., 2013).

Maccaria et al. indicated that in heroin addicts compared to the control group, TG increased while TC and HDL decreased (Maccaria et al., 1991). Furthermore, the frequency of lower cholesterolemia and low HDL were higher in the addicted group than the control. The linear regression analysis showed an inverse correlation between TC and alanine aminotransferase (ALT), an enzyme found mostly in liver damaged cells. Accordingly, the researchers suggested that lower cholesterol might be due to liver disease which is common in heroin addicts (Maccaria et al., 1991).

Unlike the above studies which demonstrated that opium addiction has no beneficial effect on plasma lipids, some data have shown the beneficial effect of opium addiction on such factors. In two studies on diabetic opium users, the level of serum TG was significantly lower than the diabetic non-users, but this was not different with respect to other lipids (Hosseini et al., 2011; Rezvanfar et al., 2011). In three studies on patients with AMI, similar results were obtained (Davoodi et al., 2005; Masoomi et al., 2008; Dehghani et al., 2013). Likewise, Fatemi et al. compared male opium addicts with male non-addicts. They observed no significant difference in TG, LDL, and HDL levels of the two groups, but the total cholesterol level was significantly less in the opium addicts (Fatemi et al., 2008). This was attributed to the lower BMI in the opium addicts. Gozashti et al. showed that TC and HDL significantly decreased in opium addicts, but TG remained unchanged (Gozashti et al., 2015). Divsalar et al. indicated that TC was significantly lower in opium and heroin addicts compared to that of the non-addicts (Divsalar et al., 2010).

The prevalence of dyslipidemia has also been reported differently in various studies, so that in some, no association was found between opium addiction and prevalence of dyslipidemia (Hosseini et al., 2011; Najafi and Sheikhvatan, 2012a; Bayani et al., 2014; Javadi et al., 2014; Najafipour et al., 2015a). In a few researches, the prevalence of dyslipidemia was lower (Maccaria et al., 1991; Dehghani et al., 2013; Rahimi et al., 2014), and in some others, the prevalence was higher in opium addicts (Aghadavoudi et al., 2015). Overall, although opium may reduce blood lipids in some cases, the majority of human studies have demonstrated that opium consumption either is ineffective or has unfavorable impact on serum lipids.

### Animal studies

In comparison to clinical studies, more consistency exists among data of animal studies. Accordingly, in most of instances, opiates did not have any useful impact on plasma lipids (Table 2). In a study on STZ-induced diabetic rats, the lipid profiles were similar in the opium treated and the control groups (Sadeghian et al., 2009). In addition, in another study on hamsters, opium administration had no significant influence on plasma lipids; however, opium augmented the effect of alcohol in increasing TC, TG, LDL, VLDL, liver enzymes, and atherogenic index. Opium raised some oxidative stress indices as well (Mohammadi et al., 2013). In another study on rats fed with cholesterol enriched diet, the implantation of a morphine pellet (75 mg) for a 5-days duration raised serum levels of TC, LDL, VLDL, and aortic cholesterol content, and it decreased HDL level. Moreover, in normal diet rats, morphine elevated TC, LDL, and VLDL. All of these effects were prevented by application of opioid antagonist naltrexone (Bryant et al., 1987). In accordance with these data, the oral treatment of mice by morphine during a 28-days period increased liver, aorta and plasma TC levels (Bryant et al., 1988). In another study, hypercholesterolaemic rabbits were exposed to opium smoke for a short period (3 days) and a longer period (28 days). Of these schedules, long term opium smoking diminished the HDL level and caused a trend of increase in LDL (Najafipour et al., 2010). In one study on rats, acute morphine administration resulted in significant increase of the level of plasma TG compared to the control group, but the other lipids were unchanged (Al Sagair, 2005). Mami et al. observed that the oral opium treatment of rabbits for 2 months elevated serum levels of TC, TG, LDL and liver enzymes compared to the control group in spite of the fact that weight gaining and abdominal fat were lower in the opium addicts (Mami et al., 2011). The data of another study showed that opium consumption in rabbits fed with cholesterol-rich diet (for 3 months), significantly intensified the impact of high cholesterol diet on the increase of plasma levels of TG, TC, and LDL. Yet, in the addicted rabbits without cholesterol-rich diets, it decreased the plasma concentrations of TC and LDL (Mohammadi et al., 2009). In another study conducted by Mohammadi et al., it was shown that the mice fed with opium for 1 month had significantly lower total cholesterol compared to the control group, but the two groups had no difference with respect to other lipids (Mohammadi et al., 2012).

## The impact of opium on blood pressure and hypertension

### Human studies

In human studies (Table 3), the intravenous injection of morphine via central sympathetic inhibition (sympatholysis) produced vasodilatation (Mansour et al., 1970), but in some studies, there was no significant difference between opium dependents and non-dependents with respect to blood pressure (Roohafza et al., 2013; Aghadavoudi et al., 2015). Furthermore, in a study on diabetic patients undergoing coronary angiography before matching of opium users with non-users for age, sex, and smoking status, the results showed that systolic and diastolic blood pressure were lower in the opium users. Yet after matching, no significant differences was observed between the two groups (Hosseini et al., 2011).

Table 3**The studies indicating the effect of opium on blood pressure and hypertension**.

**Table:** 

**A—HUMAN STUDIES**							
**Study reference**	**Type of Substance and duration of usage**	**Type of study**	**Study population**	**Underlying disease**	**Results (addicts compared to non-addicts)**		
					**BMI**	**BP**	**Prevalence of hypertension**
Roohafza et al., 2013	Opium addicted	Cohort	*n* = 569 men (99 opium addicts, 470 non-addicts)	AMI	↔	↔	ND
Hosseini et al., 2011	Opium	Cross-sectional	*n* =456 men/women (228 opium addict, 228 non-addict)	Diabetes mellitus	↔	↔	↔
Aghadavoudi et al., 2015	Opium (for 12.6 ± 7.7 years)	Cross-sectional	*n* = 325 men/women (117 opium abuser, 208 non-abuser)	CAD	↓	↔	↓
Rahimi et al., 2014	Opium addicted	Cross-sectional	*n* = 374 men/women (179 opium user, 195 non-user)	Diabetes mellitus	↓	↑	↔
Masoomi et al., 2015	Opium addicted	Cross-sectional	*n* = 217 men (103 opium addict, 114 non-addict)	None	ND	↓	↔
Mansour et al., 1970	Morphine (15 mg/kg single dose)	Inter-ventional	Not reported	None	ND	↓	ND
Yousefzadeh et al., 2015	Opium (for 11.8 years)	Cross-sectional	*n* = 5332 men/women (811 current opium users, 176 former opium users. 4340 non-user)	None	↓	ND	↑
Bayani et al., 2014	Opium (for more Than 6 months)	Cross-sectional	*n* = 97 men/women (48 opium addicts, 49 non-addicts)	ACS + type 2 diabetes	↔	ND	↔
Najafi and Sheikhvatan, 2012a	Opium addicted	Cross-sectional	*n* = 232 (26 opium addicts, 206 non-addicts)	advanced CAD+ type 2 diabetes	↓	ND	↔
Javadi et al., 2014	Opium addicted	Cross-sectional	*n* = 304 men/women (152 opium user, 152 non-user)	AMI	ND	ND	↔
Najafipour et al., 2015a	Opium addicted	Cross-sectional	*n* = 5896 men/women (non-users, occasional users, dependent users)	None	↔	ND	↔
Davoodi et al., 2005	Opium	Cohort	*n* = 160 (45 opium-dependent, 115 non-dependent)	AMI	ND	ND	↔
Shirani et al., 2010	Opium addicted	Cross-sectional	*n* =939 men/women(opium addicts, and non-addicts)	CAD	↓	ND	↓
Masoomi et al., 2008	Opium	Cross-sectional	*n* = 240 men/women (126 opium addict, 114 non-addict)	AMI	ND	ND	↔
Dehghani et al., 2013	Opium	Cross-sectional	*n* = 460 men/women (239 opium addict, 221 non-addict)	AMI	ND	ND	↓
Najafi and Sheikhvatan, 2012b	Opium addicted	Cross-sectional	*n* = 268 men/women (38 opium addict, 230 non-addict)	CAD	↓	ND	↔

**Table:** 

**B—ANIMAL STUDIES**					
**Study reference**	**Animal**	**Type of substance and duration of usage**	**Study population**	**Underlying disease**	**Results (drug recipients compared to themselves or non-recipients)**
					**Blood pressure**
Fennessy and Rattray, 1971	Rat	Morphine (IV injection of different doses repeatedly at 20 min intervals)	*n* =48 male/female rats	None	↓
Bądzyńska, Lipkowski and Sadowski, 2016	Rat	IV infusion of Morphine (1.5 mg/kg/h) or biphalin (150 μg/kg/h) for 30 min	*n* = 36 male (normotensive, spontaneously hypertensive rats)	Spontaneously hypertensive	↓
Feldberg and Wei, 1986	Cat	Morphine injection (SC or into some regions of brain)	*n* = 14 male/female cats	None	↓
Jimenez and Fuentes, 1993	Rat	Morphine (orally for 15 days)	*n* =26 male (group housed + vehicle, isolated + vehicle, group housed + morphine, isolated + morphine)	Isolation stress	↔

*Symbols: ↓, decrease; ↑, increase; ↔, no difference*.

BP, blood pressure; ND, not detected.

The data on the prevalence of hypertension are different (Table 3). Most studies indicated that opium addiction had no significant relation with the rate of hypertension (Davoodi et al., 2005; Masoomi et al., 2008, 2015; Hosseini et al., 2011; Najafi and Sheikhvatan, 2012a,b; Roohafza et al., 2013; Bayani et al., 2014; Javadi et al., 2014; Rahimi et al., 2014). In addition, in a more recent population-based study by the authors of this article on 5900 adult individuals, opium use had no significant association with hypertension in either occasional or dependent users (Najafipour et al., 2014, 2015a). One study revealed that hypertension was significantly more prevalent in opium users than non-users (Yousefzadeh et al., 2015) while some studies showed the opposite results (Shirani et al., 2010; Dehghani et al., 2013; Aghadavoudi et al., 2015).

### Animal studies

In anesthetized rats, intravenous injection of the first dose of morphine (0.025–100 mg/kg) led to fall in their blood pressure, which was transient, and BP immediately returned to near normal (Table 3). This reduction was ascribed both to vagal bradycardia and the decrease in sympathetic tone. After the first and the second injection however, in dose of 100 mg/kg, the acute phase was followed by slow falling of BP (Fennessy and Rattray, 1971). In another study on spontaneously hypertensive rats, the infusion of morphine lessened BP via vasodilatation. The response was reversed by the injection of naloxone methiodide. It was shown that the effectis mediated by peripheral opioid receptors (Bądzyńska et al., 2016). Furthermore, in anesthetized dogs, intravenous administration of two opioid drugs (fentanyl and dextromoramide) resulted in hypotension and bradycardia, which were attributed to a reduced sympathetic tone (Laubie et al., 1974). Furthermore, the subcutaneous injection of morphine in cats or injection in to cisterna magna of their brain caused hypotension and bradycardia due to the inhibition of sympathetic tone (Feldberg and Wei, 1986). However, the rats treated by Intra-cerebroventricular (i.c.v) infusion of morphine or morphine-6-glucuronide showed no change in their arterial blood pressure (Molina et al., 1994). In one study, oral chronic treatment of rats with morphine prevented the elevation of blood pressure subsequent to social deprivation-induced stress. The authors suggested that chronic treatment by morphine caused the desensitization of opioid receptors (Jimenez and Fuentes, 1993). Conversely, in conscious rabbits, the intravenous administration of morphine raised BP via increasing sympathetic activity and serum adrenaline. These effects were blocked by naloxone (May et al., 1988). In a study on rabbits, short (3 days) and long-term (4 weeks) exposure of the animals to opium smoke caused a continuous trend of increase in blood pressure during 4 weeks (Najafipour et al., 2010).

## The mechanisms of action

**Figure 1:**
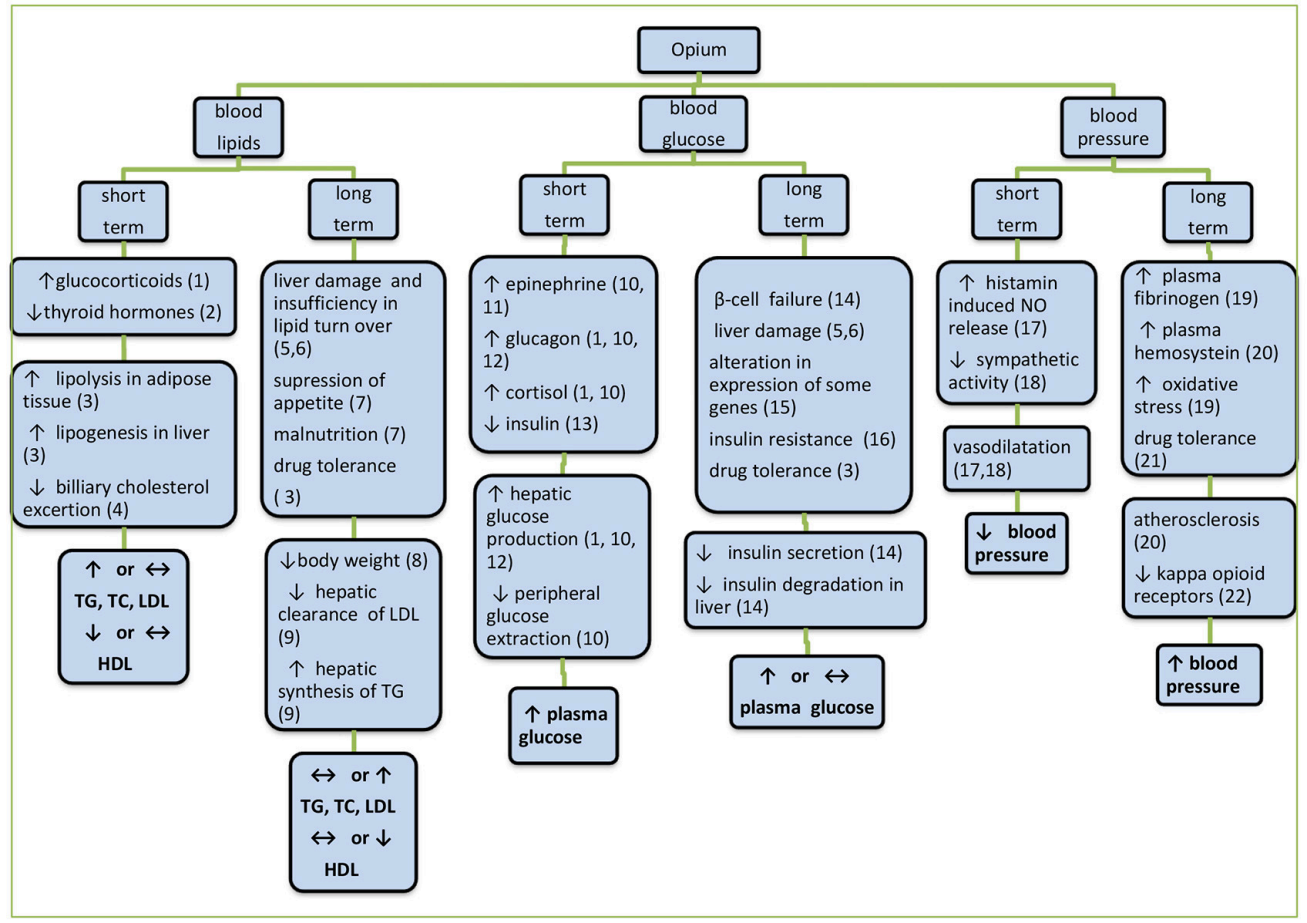
**The mechanisms proposed so far for the action of opium on blood glucose, serum lipids, and blood pressure**. Herein, short term effects are mostly exerted via hormonal and neural mechanisms but long term effects are often due to structural and functional alterations. In long-term periods depending on the dose and duration of use and other factors, different mechanisms are gradually involved. ^1^Molina et al. (1994), ^2^Berglund et al. (1990), ^3^Al Sagair (2005), ^4^Bryant et al. (1987), ^5^Payabvash et al. (2006), ^6^Zhang et al. (2004), ^7^Divsalar et al. (2010), ^8^Rahimi et al. (2014), ^9^Bryant et al. (1988), ^10^Radosevich et al. (1984), ^11^May et al. (1988), ^12^Ipp et al. (1980), ^13^Molina et al. (1994), ^14^Zandomeneghi et al. (1988), ^15^Liu and Cheng (2011), ^16^Sheldon and Quin (2005), ^17^Headrick et al. (2012), ^18^Mansour et al. (1970), ^19^Asgary et al. (2008), ^20^de Luis et al. (2005), ^21^Jimenez and Fuentes (1993), ^22^Bolte et al. (2009).

The mechanisms proposed so far for the action of opium on the three above biochemical factors are summarized in Figure 1. Short term effects are mostly exerted via hormonal and neural mechanisms, but long term effects are often due to the structural and functional alterations. In long term, depending on consumption dose and duration and other factors, different mechanisms are gradually involved. As it was mentioned before, acute hyperglycemic effects of morphine were mediated through CNS, leading to glycogenolysis and decrease of insulin release (Molina et al., 1994). This response is exaggerated in diabetic states that have been attributed to the increase of glucagon secretion (Ipp et al., 1980). In addition, insulin resistance with opiate use may be coupled with β-cell dysfunction. It has been shown that in response to an intravenous glucose load, opiate addicts had a 42% lower insulin response, accompanied by an 80% lower glucose reduction rate than non-addicted subjects (Ceriello et al., 1987). Moreover, increased fasting insulin levels in addicted subjects were also observed. In addition elevation of glucose utilization and the reduction of hepatic gluconeogenesis subsequent to the activation of peripheral opioid μ-receptors and the modification of genes involved in glucose metabolism (Liu and Cheng, 2011) are among suggested mechanisms. The reduction of 2-h post prandial glucose (2HPP) in diabetic addicted individuals (Azod et al., 2008) might be due to the decrease of gastric emptying due to opioid μ-receptor activation and thereafter delaying the intestinal glucose absorption. Moreover, the presence of underlying diseases and probable effects of medicines taken by the subjects may also come into play. In some reports despite normal glucose levels in opium addicts, there were hyperinsulinemia along with altered glucose metabolism similar to what occurs in type 2 diabetes (Sheldon and Quin, 2005). In animals however, the mechanisms such as increment of adrenalin, noradrenaline, cortisol, glucagon, etc., have been involved in increasing blood glucose (Radosevich et al., 1984). The lower FBS reported in some studies in addicted individuals has been ascribed to the anorexia secondary to opium use that cause decrease in body mass index (BMI) and insulin resistance.

Regarding the effect on blood pressure, morphine is a vasodilator in human although not through opioid receptors (OPRs), but through histamine-modulation of nitric oxide (NO) release (Headrick et al., 2012). This response is also dysregulated in diabetes (Afshari et al., 2009). Kappa-OPR agonist mediated reduction in systolic pressure has also been reported to be reduced in chronic hypertension (Bolte et al., 2009). This seems to be OPR- and K+ channel-independent, and it is less dependent upon L-type Ca++ channel function (Guo et al., 2007). Overall, it seems that the effect of opium on blood pressure is related to the dose and duration of drug consumption. Low dose and short term opium administration often decreases blood pressure and the effect is exerted via vasodilatation and reduction of sympathetic tone. In long term, the effect of opium on the reduction of blood pressure is reduced and a trend of increase in blood pressure supervenes (Joukar et al., 2010). The increase of plasma homocysteine and fibrinogen (Masoomi et al., 2002, 2015; de Luis et al., 2005) that predisposes the addicted persons to a higher risk of blood clotting, atheroma formation and resultant vascular narrowing may involve in long term effects. Homocysteine by itself is a risk factor for developing atherosclerosis and pathological changes in vascular function, especially in diabetes (de Luis et al., 2005). Overall, it is concluded that opium not only has no ameliorating effect on hypertension, but also it is a risk factor for cardiovascular diseases.

Regarding the mechanism of the effect of opium on blood lipids, some probable mechanisms proposed are reduction of hepatic clearance of LDL cholesterol from the plasma and augmenting hepatic synthesis of triglycerides, leading to the elevated levels of total cholesterol (TC) and triglycerides (TG), respectively (Bryant et al., 1988). Since lipids can penetrate blood brain barrier, they may have a role in the CNS associated with drug addiction (Hillard, 2005). However, it is not clear whether the lower level of TC (Divsalar et al., 2010; Mohammadi et al., 2013) and TG (Hosseini et al., 2011; Rezvanfar et al., 2011) in opium addicts reported in some studies are result of opium consumption or they are cause for opium addiction. Some of the authors attributed these to the lower BMI in the opium addicts. Gozashti et al. showed that TC and HDL significantly decreased in opium addicts, but TG remained unchanged (Gozashti et al., 2015). In this regards, feeding hypercholesterolaemic rabbits with opium caused an increase in the levels of TC, TG, and LDL cholesterol whereas these variables decreased in normocholestrolaemic rabbits (Mohammadi et al., 2009). In another study by the authors on hypercholesterolaemic rabbits that were exposed to opium smoke, a fall in HDL-cholesterol was found with no significant effect on the other lipids (Najafipour et al., 2010). Therefore, the route of opium consumption (enteral vs. inhalation) might have affected the impact of opium on lipid profile. In human studies, in a recent population-based study, opium use had no significant association with abnormal level of HDL and TG (Yousefzadeh et al., 2015).

Overall the results of animal studies may be more consistent and reliable because in clinical studies, some conditions may be different between addicts and non-addicts that may affect the results. Some of these factors include:

1. Nutritional factors: Many addicts lost their jobs and had to pay extra expenses to provide their required substance. This led opium users to suffer from malnutrition and vitamin deficiency. Also, the use of opiates suppresses appetite and reduces weight in many instances (Divsalar et al., 2010). Therefore, the reduction of lipids in some studies might be due to weight loss or nutrient deficiency not owing to direct effect of opium.
2. Individual factors such as underlying diseases, psycho-social problems, physical activity level, and age range of subjects may alter the effect of opium on serum lipids. Also these conditions may alter serum biochemical variables independent of the effect of opium.
3. Factors relevant to the substance including purity and ingredients of opium, concomitant use of other substances (alcohol, cigarette, tobacco,…), duration, quantity and method of usage. Opioids have negative impact on many organs such as liver. Opioids are mainly metabolized in the liver, hence chronic consumption of the substance leads to serious liver damage. It has been demonstrated that chronic administration of morphine causes the suppression of hepatic antioxidant system, elevation of oxidant indices and induction of apoptosis in hepatocytes in mice (Zhang et al., 2004; Payabvash et al., 2006). It has also been shown that there is a direct association between the duration of heroin consumption and the severity of liver injury in heroin abusers (Zhang et al., 2004; Ilić et al., 2005). Since liver plays a pivotal role in the metabolism of lipids, chronic liver diseases are associated with significant alterations in serum lipids (Ooi et al., 2005; Ghadir et al., 2010).
4. The factors relevant to research: Many human studies have small sample sizes or are hospital-based instead of population-based. Therefore, the results may not be generalized to the whole population.

Although a large body of evidences is available from human studies regarding the impact of opium consumption on cardio-metabolic disorders, however most of these studies are cross sectional in their design. Limitations of the cross-sectional studies are simultaneous data collection concerning risk factors and outcomes which prevents an exact examination of the temporal transposition between them. In other words, it cannot be said for sure whether the higher prevalence of disorders in opium users is the result of opium consumption or the cause for opium consumption. Therefore, it is suggested that more prospective longitudinal studies with large sample sizes to be conducted to clear this temporal precedence and to identify the pathophysiological mechanisms more precisely.

## Conclusion

In spite of the fact that the results of the studies into the effect of opium on blood glucose, blood lipids and blood pressure are relatively incoherent, these findings, not only do not support the traditional beliefs about beneficial effects of opium and its derivatives on diabetes, dyslipidemia and hypertension, but they also represents their long term detrimental effects on these disorders and on many body organs. Therefore, public awareness about these destructive effects of unauthorized use of opium would have an important role in health of individuals and the society.

## Author contributions

HN contributed to the conception of the work, interpretation of data, drafting, critical revising, and final approval of the version to be published. AB contributed to the acquisition and interpretation of data, drafting and final approval of the version to be published. Both authors agree to be accountable for all aspects of the work.

## Funding

This work was supported by Kerman university of Medical sciences, Kerman Iran, Grant no 88/110.

### Conflict of interest statement

The authors declare that the research was conducted in the absence of any commercial or financial relationships that could be construed as a potential conflict of interest.

## References

[B1] Afarinesh M. R. Haghpanah T. Divsalar K. Dehyadegary E. Shaikh-Aleslami A. Mahmoodi M. (2014). Changes in serum biochemical factors associated with opium addiction after addiction desertion. Addict. Health 6, 138–145. 25984281 PMC4354219

[B2] Afshari R. Maxwell S. R. Webb D. J. Bateman D. N. (2009). Morphine is an arteriolar vasodilator in man. Br. J. Clin. Pharmacol. 67, 386–393. 10.1111/j.1365-2125.2009.03364.x 19371311 PMC2679101

[B3] Aghadavoudi O. Eizadi-Mood N. Najarzadegan M. R. (2015). Comparing cardiovascular factors in opium abusers and non-users candidate for coronary artery bypass graft surgery. Adv. Biomed. Res. 4, 1–7. 10.4103/2277-9175.148294 25625118 PMC4300596

[B4] Akif Q. Nakhshab C. Sadia M. Muhammad F. U. H. Nadia A. (2013). Evaluation of lipids and lipoprotein levels in opium and heroin addicts in Punjabi population. Esculapio 9, 163–167.

[B5] Al Sagair O. A. (2005). Effect of morphine sulphate on total lipids and triglycerides contents in serum and brain regions of rat. Med. Islamic World Sci. 15, 117–125.

[B6] Asadi K. G. Rashidinejad H. R. Aghaee M. M. Ahmadi J. Rahmani M. R. Mahmoodi M.. (2008). Opium can differently alter blood glucose, sodium and potassium in male and female rats. Pak. J. Pharm. Sci. 21, 180–184. 18390449

[B7] Asgary S. Sarrafzadegan N. Naderi G. A. Rozbehani R. (2008). Effect of opium addiction on new and traditional cardiovascular risk factors: do duration of addiction and route of administration matter? Lipids Health Dis. 7, 1–5. 10.1186/1476-511x-7-42 18980684 PMC2588593

[B8] Azod L. Rashidi M. Afkhami-Ardekani M. Kiani G. Khoshkam F. (2008). Effect of opium addiction on diabetes. Am. J. Drug Alcohol Abuse 34, 383–388. 10.1080/00952990802122580 18584567

[B9] Bądzyńska B. Lipkowski A. W. Sadowski J. (2016). An antihypertensive opioid: Biphalin, a synthetic non-addictive enkephalin analog decreases blood pressure in spontaneously hypertensive rats. Pharmacol. Rep. 68, 51–55. 10.1016/j.pharep.2015.06.006 26721351

[B10] Bayani M. Nazemi S. Khosoosi Niaki M. R. Ramezani M. Khani A. (2014). Opium consumption and lipid and glucose parameters in diabetic patients with acute coronary syndrome; a survey in northern Iran. Tunis. Med. 92, 496–500. 25775291

[B78] Berglund L. A. Millard W. J. Gabriel S. M. Simpkins J. W. (1990). Opiate-thyroid hormone interactions in the regulation of thyrotropin secretion in the rat. Neuroendocrinology 52, 303–308. 10.1159/000125602 2120610

[B11] Bolte C. Newman G. Scultzjel J. (2009). Hypertensive state, independent of hypertrophy, exhibits an attenuated decrease in systolic function on cardiac k-opioid receptor stimulation. Am. J. Physiol. Heart Circ. Physiol. 296, H967–H975. 10.1152/ajpheart.00909.2008 19181965 PMC2670690

[B12] Bryant H. U. Story J. A. Yim G. K. W. (1987). Morphine-induced alterations in plasma and tissue cholesterol levels. Life Sci. 41, 545–554. 10.1016/0024-3205(87)90406-1 3600193

[B13] Bryant H. U. Story J. A. Yim G. K. W. (1988). Stress and morphine-induced elevations of plasma and tissue cholesterol in mice: reversal by naltrexone. Biochem. Pharmacol. 37, 3777–3781. 10.1016/0006-2952(88)90415-7 3178891

[B14] Ceriello A. Giugliano D. Passariello N. Quatraro A. Dello Russo P. Torella R.. (1987). Impaired glucose metabolism in heroin and methadone users. Horm. Metabol. Res. 19, 430–433. 10.1055/s-2007-1011844 3319862

[B15] Davoodi G. Sadeghian S. Akhondzadeh S. Darvish S. Alidoosti M. Amirzadegan A. (2005). Comparison of specifications, short-term outcome and prognosis of acute myocardial infarction in opium dependent patients and non-dependents. German J. Psychiatry 8, 33–37.

[B16] Dehghani F. Masoomi M. Haghdoost A. K. (2013). Relation of opium addiction with the severity and extension of myocardial infarction and its related mortality. Addict. Health 5, 35–42. 24494156 PMC3905566

[B17] de Luis D. A. Fernandez N. Arranz M. L. Aller R. Izaola O. Romero E. (2005). Total homocysteine levels relation with chronic complications of diabetes, body composition, and other cardiovascular risk factors in a population of patients with diabetes mellitus type 2. J. Diabetes Complicat. 19, 42–46. 10.1016/j.jdiacomp.2003.12.003 15642489

[B18] Divsalar K. Haghpanah T. Afarinesh M. (2010). Opium and heroin alter biochemical parameters of human's serum. Am. J. Drug Alcohol Abuse 36, 135–139. 10.3109/00952991003734277 20465370

[B19] European Food Safety Authority (EFSA) (2011). Scientific opinion on the risks for public health related to the presence of opium alkaloids in poppy seeds. EFSA J. 9:2405 10.2903/j.efsa.2011.2405

[B20] Farahani M. A. Mohammadi E. Ahmadi F. Maleki M. Hajizadeh E. (2008). Cultural barriers in the education of cardiovascular disease patients in Iran. Int. Nurs. Rev. 55, 360–366. 10.1111/j.1466-7657.2008.00635.x 19522955

[B21] Fatemi S. S. Hasanzadeh M. Arghami A. Sargolzaee M. R. (2008). Lipid profile comparison between opium addicts and non-addicts. J. Teh. Univ. Heart Center 3, 169–172.

[B22] Feldberg W. Wei E. (1986). Analysis of cardiovascular effects of morphine in the cat. Neuroscience 17, 495–506. 10.1016/0306-4522(86)90262-9 3703247

[B23] Fennessy M. R. Rattray J. F. (1971). Cardiovascular effects of intravenous morphine in the anaesthetized rat. Eur. J. Pharmacol. 14, 1–8. 10.1016/0014-2999(71)90116-6 4396659

[B24] Ghadir M. R. Riahin A. K. Havaspour A. Nooranipour M. Habibinejad A. A. (2010). The relationship between lipid profile and severity of liver damage in cirrhotic patients. Hepat. Mon. 10, 285–288. 22312394 PMC3271321

[B25] Gozashti M. H. Yazdi F. Salajegheh P. Dehesh M. M. Divsalar K. (2015). Fasting blood glucose and insulin level in opium addict versus non-addict individuals. Addict. Health 7, 54–59. 26322211 PMC4530194

[B26] Guo H. T. Zhang R. H. Huang L. Y. Li J. Liu Y. L. Bi H.. (2007). Mechanisms involved in the hypotensive effect of kappa-opioid receptor agonist in hypertensive rats. Arch. Med. Res. 38, 723–729. 10.1016/j.arcmed.2007.04.009 17845890

[B27] Headrick J. P. Pepe S. Peart J. N. (2012). Non-analgesic effects of opioids: cardiovascular effects of opioids and their receptor system. Curr. Pharm. Des. 18, 6090–6100. 10.2174/138161212803582360 22747541

[B28] Hillard C. J. (2005). Lipids and drugs of abuse. Life Sci. 77, 1531–1542. 10.1016/j.lfs.2005.05.004 15946699

[B29] Hosseini S. K. Masoudkabir F. Vasheghani-Farahani A. Alipour-Parsa S. Sheikh Fathollahi M. Rahimi-Foroushani A.. (2011). Opium consumption and coronary atherosclerosis in diabetic patients: a propensity score-matched study. Planta Med. 77, 1870–1875. 10.1055/s-0031-1280017 21800277

[B30] Ilić G. Karadžić R. Kostić-Banović L. Stojanović J. (2005). Chronic intravenous heroin abuse: impact on the liver. Med. Biol. 12, 150–153.

[B31] Ipp E. Schusdziarra V. Harris V. Unger R. H. (1980). Morphine-induced hyperglycemia: role of insulin and glucagon. Endocrinology 107, 461–463. 10.1210/endo-107-2-461 6993188

[B32] Jafari S. Rahimi Movaghar A. Craib K. Baharlou S. Mathias R. (2009). Socio-cultural factors associated with the initiation of opium use in Darab, Iran. Int. J. Ment. Health Addict. 7, 376–388. 10.1007/s11469-008-9176-y

[B33] Javadi H. R. Allami A. Mohammadi N. Alauddin R. (2014). Opium dependency and in-hospital outcome of acute myocardial infarction. Med. J. Islam. Repub. Iran 28, 2–7. 25679001 PMC4313445

[B34] Jimenez I. Fuentes J. A. (1993). Subchronic treatment with morphine inhibits the hypertension induced by isolation stress in the rat. Neuropharm 32, 223–227. 10.1016/0028-3908(93)90104-B 8474618

[B35] Joukar S. Najafipour H. Mirzaeipour F. Nasri H. R. (2010). The effect of passive opium smoking on cardiovascular indices of rabbits with normal and ischemic hearts. Open Cardiovasc. Med. J. 4, 1–6. 10.2174/1874192401004010001 20148098 PMC2817881

[B36] Kalant H. (1997). Opium revisited: a brief review of its nature, composition, non-medical use and relative risks. Addiction 92, 267–277. 10.1046/j.1360-0443.1997.9232673.x 9219389

[B37] Karam G. A. Reisi M. Kaseb A. A. Khaksari M. Mohammadi A. Mahmoodi M.. (2004). Effects of opium addiction on some serum factors in addicts with non-insulin-dependent diabetes mellitus. Addict. Biol. 9, 53–58. 10.1080/13556210410001674095 15203439

[B38] Laubie M. Schmitt H. Canellas J. Roquebert J. Demichel P. (1974). Centrally mediated bradycardia and hypotension induced by narcotic analgesics: dextromoramide and fentanyl. Eur. J. Pharmacol. 28, 66–75. 10.1016/0014-2999(74)90113-7 4154206

[B39] Liu I. M. Cheng J. T. (2011). Mediation of endogenous β-endorphin in the plasma glucose-lowering action of herbal products observed in type1-like diabetic rats. Evid. Based Complement. Altern. Med. Article ID 987876, 10 pages. 10.1093/ecam/nen078 19095661 PMC3147137

[B40] Maccaria S. Bassia C. Zanoni P. Plancher A. C. (1991). Plasma cholesterol and triglycerides in heroin addicts. Drug Alcohol Depend. 29, 183–187. 10.1016/0376-8716(91)90047-3 1797528

[B41] Mahmoodi M. Hosseini-Zijoud S.-M. Hosseini J. Sayyadi A. R. Hajizadeh M. R. Hassanshahi G. (2012). Opium withdrawal and some blood biochemical factors in addicts' individuals. Adv. Biol. Chem. 2, 167–170. 10.4236/abc.2012.22020

[B42] Mami S. Eghbali M. Cheraghi J. Mami F. Pourmahdi B. M. Salati A. P. (2011). Effect of opium addiction on some serum parameters in rabbit. Global Vet. 7, 310–314.

[B43] Mansour E. Capone R. Mason D. T. Amsterdam E. A. Zellis R. (1970). The mechanism of morphine-induced peripheral arteriolar dilation-central nervous sympatholysis. Am. J. Cardiol. 26:648 10.1016/0002-9149(70)90503-5

[B44] Masoomi M. Azdaki N. Shahouzehi B. (2015). Elevated plasma homocysteine concentration in opium-addicted individuals. Addict. Health 7, 1–8. 26885351 PMC4741235

[B45] Masoomi M. Ghaemi F. Haghdoost A. K. Rashidinejad H. R. (2008). ST segment resolution in opium addict patients after thrombolytic therapy for acute myocardial infarction. ARYA Atheros. J. 4, 103–107.

[B46] Masoomi M. Nasri H. Farajpour F. (2002). Comparison of plasma fibrinogen level in opium addict men with non-addict men. J. Kerman Univ. Med. Sci. 9, 27–31. [in Persian].

[B47] Masoudkabir F. Sarrafzadegan N. Eisenberg M. J. (2013). Effects of opium consumption on cardiometabolic diseases. Nat. Rev. Cardiol. 10, 733–740. 10.1038/nrcardio.2013.159 24145895

[B48] May C. N. Ham I. W. Heslop K. E. Stone F. A. Mathias C. J. (1988). Intravenous morphine causes hypertension, hyperglycaemia and increases sympatho-adrenal outflow in conscious rabbits. Clin. Sci. 75, 71–77. 10.1042/cs0750071 3409626

[B49] Mohammadi A. Abbasi Oshaghi E. Sorkhani A. Oubari F. Hosseini Kia R. Rezaei A. (2012). Effect of opium on lipid profile and expression of liver X receptor alpha (LXRα) in normolipidemic mouse. Food Nutr. Sci. 3, 249–254. 10.4236/fns.2012.32036

[B50] Mohammadi A. Darabi M. Nasry M. Saabet-Jahromi M. J. Malekpour Afshar R. Sheibani H.. (2009). Effect of opium addiction on lipid profile and atherosclerosis formation in hypercholesterolemic rabbits. Exp. Toxicol. Pathol. 61, 145–149. 10.1016/j.etp.2008.08.001 18838257

[B51] Mohammadi A. Mirzaei F. Jamshidi M. Yari R. Pak S. Noori Sorkhani A. (2013). The *in vivo* biochemical and oxidative changes by ethanol and opium consumption in Syrian hamsters. Int. J. Biol. 5, 14–22. 10.5539/ijb.v5n4p14

[B52] Molina P. E. Hashiguchi Y. Ajmal M. Mazza M. Abumrad N. N. (1994). Differential hemodynamic, metabolic and hormonal effects of morphine and morphine-6-glucuronide. Brain Res. 664, 126–132. 10.1016/0006-8993(94)91962-3 7895021

[B53] Najafi M. Sheikhvatan M. (2012a). Plausible impact of dietary habits on reduced blood sugar in diabetic opium addicts with coronary artery disease. Int. Cardiovasc. Res. J. 6, 75–78. 24757596 PMC3987411

[B54] Najafi M. Sheikhvatan M. (2012b). Does analgesic effect of opium hamper the adverse effects of severe coronary artery disease on quality of life in addicted patients? Anesth. Pain 2, 22–27. 10.5812/aapm.5139 24223329 PMC3821107

[B55] Najafipour H. Joukar S. Malekpour-Afshar R. Mirzaeipour F. Nasri H. R. (2010). Passive opium smoking does not have beneficial effect on plasma lipids and cardiovascular indices in hypercholesterolemic rabbits with ischemic and non-ischemic hearts. J. Ethnopharmacol. 127, 257–263. 10.1016/j.jep.2009.11.011 19914364

[B56] Najafipour H. Masoomi M. Shahesmaeili A. Haghdoost A. A. Afshari M. Nasri H. R.. (2015a). Effects of opium consumption on coronary artery disease risk factors and oral health: results of kerman coronary artery disease risk factors study a population-based survey on 5900 subjects aged 15-75 years. Int. J. Prev. Med. 6, 42. 10.4103/2008-7802.157470 26097671 PMC4455126

[B57] Najafipour H. Nasri H. R. Afshari M. Moazenzadeh M. Shokoohi M. Foroud A.. (2014). Hypertension: diagnosis, control status and its predictors in general population aged between 15 and 75 years:a community-based study in southeastern Iran. Int. J. Public Health 59, 999–1009. 10.1007/s00038-014-0602-6 25227395

[B58] Najafipour H. Sanjari M. Shokoohi M. Haghdoost A. A. Afshari M. Shadkam M.. (2015b). Epidemiology of diabetes mellitus, pre-diabetes, undiagnosed and uncontrolled diabetes and its predictors in general population aged 15 to 75 years: a community-based study (KERCADRS) in Southeastern Iran. J. Diabetes 7, 613–621. 10.1111/1753-0407.12195 25042896

[B59] Ooi K. Shiraki K. Morishita Y. Nobori T. (2005). Clinical significance of abnormal lipoprotein patterns in liver diseases. Int. J. Mol. Med. 15, 655–660. 10.3892/ijmm.15.4.655 15754028

[B60] Payabvash S. Beheshtian A. Hassanzadeh S. A. Kiumehr S. Ghahremani M. H. Tavangar S. M.. (2006). Chronic morphine treatment induces oxidant and apoptotic damage in the mice liver. Life Sci. 79, 972–980. 10.1016/j.lfs.2006.05.008 16750225

[B61] Radosevich P. M. Williams P. E. Lacy D. B. McRae J. R. Steiner K. E. Cherrington A. D.. (1984). Effects of morphine on glucose homeostasis in the conscious dog. J. Clin. Invest. 74, 1473–1480. 10.1172/JCI111560 6148357 PMC425317

[B62] Rahimi N. Gozashti M. H. Najafipour H. Shokoohi M. Marefati H. (2014). Potential effect of opium consumption on controlling diabetes and some cardiovascular risk factors in diabetic patients. Addict. Health 6, 1–6. 25140211 PMC4137437

[B63] Rezvanfar M. R. Farahany H. Rafiee M. Kaboli S. (2011). Opium consumption challenge and diabetes mellitus control. Iran J. Diabetes Obes. 3, 72–76.

[B64] Roohafza H. Talaei M. Sadeghi M. Haghani P. Shokouh P. Sarrafzadegan N. (2013). Opium decreases the age at myocardial infarction and sudden cardiac death: a long- and short-term outcome evaluation. Arch. Iran Med. 16, 154–160. 23432167

[B65] Sadava D. Alonso D. Hong H. Pettit-Barrett D. (1997). Effect of methadone addiction on glucose metabolism in rats. Gen. Pharmacol. 28, 27–29. 10.1016/S0306-3623(96)00165-6 9112073

[B66] Sadeghian S. Boroumand M. A. Sotoudeh-Anvari M. Rabbani S. Sheikh Fathollahi M. Abbasi A. (2009). Effect of opium on glucose metabolism and lipid profiles in rats with streptozotocin-induced diabetes. Pol. J. Endocrinol. 60, 258–262. 19753539

[B67] Sanli D. B. Bilici R. Suner O. Citak S. Kartkaya K. Mutlu F. S. (2015). Effect of different psychoactive substances on serum biochemical parameters. Int. J. High Risk Behav. Addict. 4, 1–5. 10.5812/ijhrba.22702 26405680 PMC4579556

[B68] Schiff P. L. (2002). Opium and its alkaloids. Am. J. Pharm. Educ. 66, 186–194. 845809

[B69] Sheldon B. H. Quin J. D. (2005). Diabetes and illicit drug use. Pract. Diabetes Int. 22, 222–224. 10.1002/pdi.821

[B70] Shirani S. Shakiba M. Soleymanzadeh M. Esfandbod M. (2010). Can opium abuse be a risk factor for carotid stenosis in patients who are candidates for coronary artery bypass grafting? Cardiol. J. 17, 254–258. 20535715

[B71] Salman T. M. El Zahaby M. M. Mansour O. A. Omran G. A. Gomma S. M. Gad H. S. (2010). Oxidative stress and lipotoxicity of bhang and opium addiction. Effects on adrenal gland secretions. Dyn. Biochem. Proc. Biotechnol. Mol. Biol. 4, 50–54.

[B72] United Nations Office on Drugs and Crime (2014). World Drug Report. Available online at: https://www.unodc.org/documents/wdr2014/World_Drug_Report_2014_web.pdf (Accessed June 2014).

[B73] United Nations Office on Drugs and Crime (2015). World Drug Report. Available online at: http://reliefweb.int/report/world/unodc-world-drug-report-2015 (Accessed May 2015).

[B74] Vuong C. Van Uum S. H. M. O'Dell L. E. Lutfy K. Friedman T. C. (2010). The effects of opioids and opioid analogs on animal and human endocrine systems. Endocrine Rev. 31, 98–132. 10.1210/er.2009-0009 19903933 PMC2852206

[B75] Yousefzadeh G. Shokoohi M. Najafipour H. Eslami M. Salehi F. (2015). Association between opium use and metabolic syndrome among an urban population in Southern Iran: results of the Kerman Coronary Artery Disease Risk Factor Study (KERCADRS). ARYA Atheroscler. 11, 14–20. 26089926 PMC4460348

[B76] Zandomeneghi R. Luciani A. Massan M. Montanan P. Pavesi C. (1988). Effects of heroin addiction on the responses of glucose, C-peptide and insulin to a standard meal. Clin. Sci. 74, 283–288. 10.1042/cs0740283 3278831

[B77] Zhang Y. T. Zheng Q. S. Pan J. Zheng R. L. (2004). Oxidative damage of biomolecules in mouse liver induced by morphine and protected by antioxidants. Basic Clin. Pharmacol. Toxicol. 95, 53–58. 10.1111/j.1742-7843.2004.950202.x 15379780

